# GENETIC RISK, MODIFIABLE FACTORS, AND COGNITIVE PERFORMANCE IN MIDDLE- AND OLDER-AGED CANADIANS

**DOI:** 10.1093/geroni/igae098.3484

**Published:** 2024-12-31

**Authors:** Shawna Hopper, Cindy Barha, Mari DeMarco, Theodore Cosco, Andrew Wister, John Best

**Affiliations:** Simon Fraser University, Vancouver, British Columbia, Canada; University of Calgary, Calgary, Alberta, Canada; University of British Columbia, Vancouver, British Columbia, Canada; Simon Fraser University, Vancouver, British Columbia, Canada; Simon Fraser University, Vancouver, British Columbia, Canada; Simon Fraser University, Vancouver, British Columbia, Canada

## Abstract

Whether the apolipoprotein E (APOE) e4 allele interacts with modifiable factors to predict cognitive performance is unclear. This study examined 26,448 participants (45 to 86 years of age at baseline) in the Canadian Longitudinal Study on Aging who had their blood genotyped for the APOE e4 allele, of whom 6,305 (24%) were e4 carriers. Cognitive outcomes were Rey Auditory Verbal Learning Test 5-minute delayed recall, Mental Alterations Test, and Animal Fluency Test, which were collected at baseline (December 2011 to July 2015), follow-up 1 (median 2.9 years after baseline), and follow-up 2 (median 5.8 years after baseline). We examined interactions between time from baseline, APOE e4 status and the following exposures: self-reported physical activity and a composite risk score comprised of sedentary behavior, smoking, depressive symptoms, social isolation, whole blood hemoglobin A1c fraction, and percent body fat. Models were adjusted for age, sex, ethnicity, study language (French/English), residential setting (urban/rural), and educational attainment. Across cognitive outcomes, an age by APOE e4 interaction was observed (p values ranging from <.001 to.01), such that the difference in cognitive performance between carriers and non-carriers increased as participant age increased. Although physical activity and composite risk were associated with cognitive performance (all p values <.01), there was not convincing evidence that these associations varied by e4 carrier status after correcting for multiple comparisons. Together, these findings confirm that APOE e4 carriers show greater reductions in cognitive performance but do not suggest that e4 status alters associations between modifiable risk and cognitive decline.

